# Improved quality of life and joint functions in patients with knee rheumatoid arthritis who underwent five portal arthroscopic synovectomy

**DOI:** 10.7717/peerj.4727

**Published:** 2018-04-30

**Authors:** Wen-Xin Liu, Yao Jiang, Qing-Xiang Hu, Xie-Bo You

**Affiliations:** 1 Department of Orthopedics, The Third Affiliated Hospital of Soochow University, Changzhou, China; 2 Department of Orthopedics, Shanghai Sixth People’s Hospital, Shanghai, China

**Keywords:** Rheumatoid arthritis, Synovectomy, Negative pressure drainage, Arthroscopy

## Abstract

**Objectives:**

To evaluate the outcomes of patients with rheumatoid arthritis (RA) of the knee who underwent five portal arthroscopic synovectomy, with or without post-operative negative pressure drainage (NPD).

**Material and Methods:**

A prospective clinical trial was performed. Patients with class I, II, and III RA of the knee were enrolled. They underwent five portal arthroscopic synovectomy. Post-operatively, they received either NPD (group A) or non-NPD (group B). Health assessment questionnaire (HAQ), disease activity score 28 (DAS 28), and Lysholm knee joint score were evaluated before the operations, and at six weeks, three months, and one year after the operations.

**Results:**

A total of 36 patients were enrolled into the study, with 63.9% (23) female patients and mean age of 47.2 years old. All of the patients had clinical symptoms (joint swelling, pain, and dysfunction) for at least six months with poor responses to the traditional pharmaceutical therapy. There were 12, 16, and eight patients in class I, II, and III RA groups, respectively (six IA, six IB, eight IIA, eight IIB, four IIIA, and four IIIB). One year after the operation, patients had statistically significant improvements on HAQ, DAS 28, and Lysholm knee joint scores. More improvements were observed in patients with class I diseases. There were no statistically significant differences between group A and B.

**Conclusion:**

Five portal arthroscopic synovectomy could increase the quality of life, decrease disease activities, and improve joint functions in patients with RA. More benefits were observed in patients with early disease developments. Patients in the NPD group did not show more improvements compared to the patients in the non-NPD group.

## Introduction

Rheumatoid arthritis (RA) is a chronic autoimmune disease, with a worldwide prevalence 1–2% in the general population (Gibofsky, 2012). The underlying pathophysiology starts with abnormal immune responses and inflammations, and then leads to B and T cell activation, as well as the release of autoimmune antibodies and various cytokines. These can lead to chronic joint inflammations, and finally result in synovial membrane infiltrations, cartilage damages, and bone erosions (McInnes & Schett, 2011). The most common joint involved is knee, which causes swelling, pain, warm, stiffness, and joint dysfunctions (Lee & Choi, 2012).

Common treatments for RA are to alleviate joint inflammations and relieve clinical symptoms. The medications used in the clinic include nonsteroidal anti-inflammatory drugs (NSAIDs), corticosteroids, and disease-modifying antirheumatic drugs (DMARDs) (Monti et al., 2015; Vivar & Van Vollenhoven, 2014). However, some of these medications could only reach partial relief or have no effects at all. Some of these medications are too expensive for long-term use (Wolfe, 1995; Caporali et al., 2009). A better treatment modality is required. Synovectomy, which removed the inflamed synovial membrane, was applied as a treatment choice for RA (Jensen et al., 1991; Lee et al., 2014). However, open synovectomy has limited clinical applications, due to its complications of long recovery time, increased risks for bleeding, and infections (Matsui et al., 1989). With the development of arthroscopy, minimally invasive arthroscopic synovectomy has become an important treatment method for rheumatoid arthritis (Klug, Wittmann & Weseloh, 2000; Momohara et al., 2001). It not only significantly reduced the severity of pain and dysfunctions, but also decreased the recurrence rate and slowed the progression of the disease (Kanbe et al., 2015; Ogilvie-Harris & Weisleder, 1995; Gibbons, Gosal & Bartlett, 2002; Roch-Bras et al., 2002). Arthroscopic synovectomy can be performed through either five or six portal approach. Previous studies have demonstrated that five portal arthroscopic synovectomy, with only five small incisions made around the knee joint, could be as effective as open synovectomy for knee synovitis from various causes (Pan et al., 2012; Kubat et al., 2010; Jain et al., 2013). The benefits of five portal arthroscopic synovectomy in the treatment of knee rheumatoid arthritis require further investigations. Meanwhile, in patients who received orthopedic surgeries, studies have shown controversial results on whether negative pressure drainage (NPD) could decrease the incident of infection, accelerate the wound healing, or improve patient outcomes (Siqueira et al., 2016; Karlakki et al., 2013; Clifton et al., 2007). Previous studies on NPD were mostly performed after meniscus or cruciate ligament operations. There were limited studies to investigate the effects of NPD after arthroscopic synovectomy in patients with RA.

In the current study, we performed arthroscopic synovectomy in patients with rheumatoid arthritis of the knee. We compared and reported their quality of life, disease activity, and joint functional outcomes before and after the operations, as well as between patients with or without post-operative NPD.

## Methods

### Study design and participants

The current study was a prospective clinical trial performed in an academic teaching hospital between January, 2014 and December, 2015. Shanghai Sixth People’s Hospital granted Ethical approval to carry out the study within its facilities (Ethical Application Ref: 0130148). All the study participants signed the informed consent. This study was registered at the Chinese Ethics Committee of Registering Clinical Trials (chiCTR-IPQ-17012440).

Inclusion criteria were: (1) rheumatoid arthritis of the knee diagnosed based on American College of Rheumatology/European League Against Rheumatism (ACR/EULAR) classification criteria for rheumatoid arthritis (Raja et al., 2012); (2) could be classified into class I, II, and III knee arthritis according to the RAX Steinbrocker classification system (Steinbrocker, Traeger & Batterman, 1949); (3) clinical symptoms (swelling, pain, and dysfunction) have persisted for at least six months with poor responses to the traditional pharmaceutical therapy; (4) unilateral knee involvement; (5) signed informed consent and agreed to receive clinical follow-up appointments and evaluations.

Exclusion criteria were: (1) cellulitis around the knee joint; (2) synovitis caused by gout or ankylosing spondylitis; (3) cancer, diabetes, or other connective tissue diseases.

### Study protocol

After informed consent process, patients were classified into class I, II, and III based on the RAX Steinbrocker classification system. Then, patients were further assigned into either A (NPD) or B (non-NPD) group according to the even or odd number of their ordered admission numbers.

All the patients received the surgical operations under the laryngeal mask general anesthesia with regional nerve (femoral nerve and sciatic nerve) blocks by one orthopedic physician. With patients in the supine position and affected leg close to the edge of the bed, cotton-lined tourniquet was placed to the thigh proximal to the affected knee. Two arthroscopic pads were placed at the lateral side of the distal thigh (close to the lateral side of joint line) and middle thigh, in order to ensure that the leg could be easily placed with a slight valgus overhanging position and knee could be placed at 90° flexion position. After standard sterilization and applying dressings to the lifted leg, the tourniquet was inflated to a pressure of 400–600 mmHg. A complete synovial resection usually required at least five portal approaches. Thus, in the current study, we used five portal approaches (high anterolateral approach, high anteromedial approach, high posterior medial approach, posterior lateral approach, and superolateral patellar approach) in order to completely resect the knee synovium.

**Figure 1:**
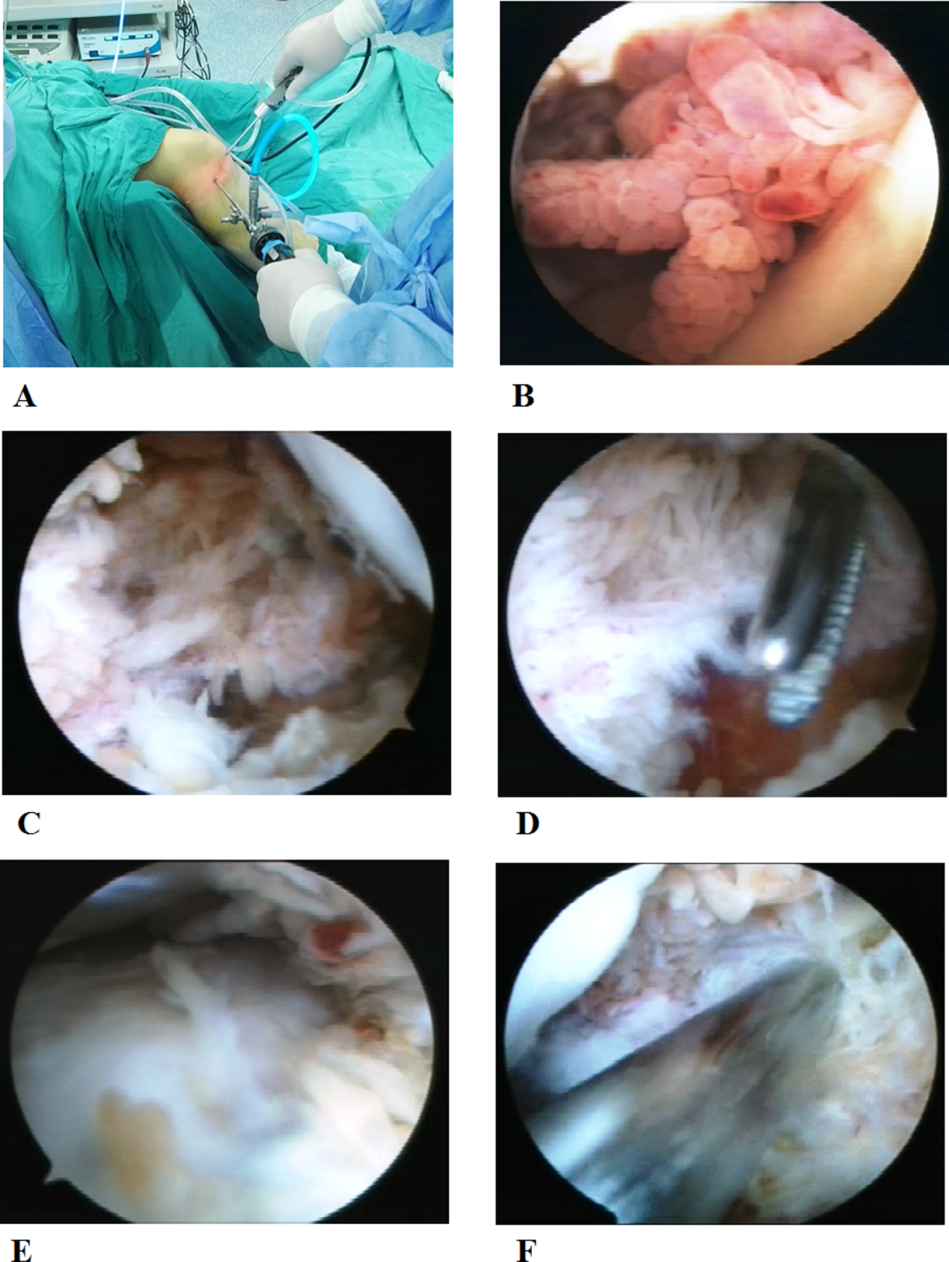
Arthroscope was at the high anterolateral portal and the shaver was at the high anteromedial portal (A). Large amount of inflamed synovial membrane was observed (B). The femoral medial condyle was visualized and large amount of inflamed synovial membrane was observed in the posteromedial compartment (C). Arthroscope was inserted through the high posteromedial portal, and then into posteromedial compartment (D). Lateral femoral condyle and lateral meniscus were visualized with inflamed synovial membrane (E). The shaver was inserted through the posterolateral portal under the direct visualization of arthroscope (F). Photographs by Wen-Xin Liu.

**Figure 2:**
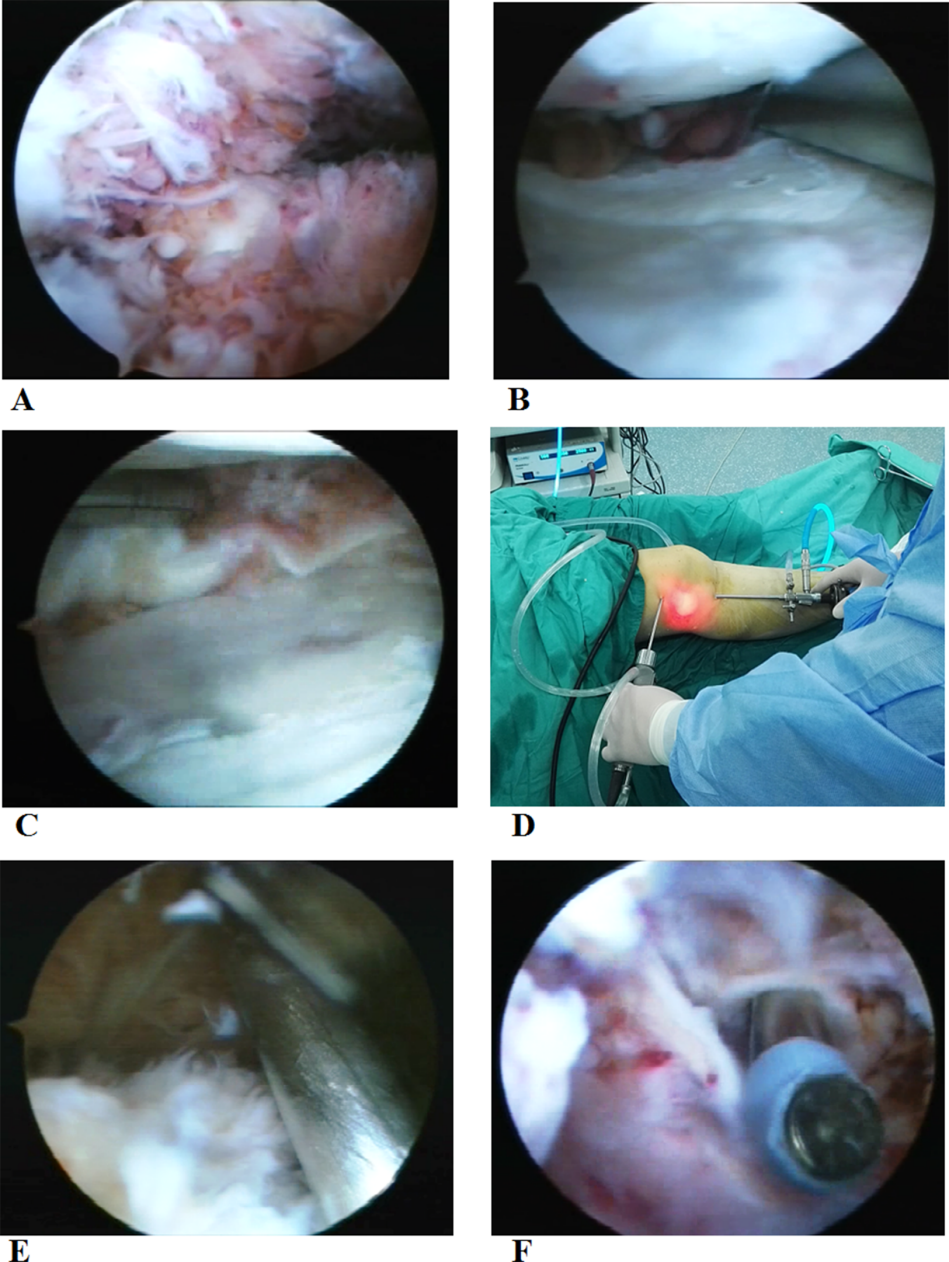
Arthroscope was at the high anterolateral portal and inflammation was found at the intercondylar fossa (A). In the anteromedial compartment, inflammation was found to the synovial membrane and the medial meniscus (B). In the anterolateral compartment, inflammation was found to the synovial membrane and lateral meniscus (C). Arthroscope was placed through the high anterolateral portal (D). The shaver was inserted through the superolateral patellar portal after needle positioning (E). A vaporized electric knife was used for complete hemostasis (F). Photographs by Wen-Xin Liu.

The surgical procedures were:

1. Initial exploration of knee joint: arthroscope (Linvatec, Largo, Florida, USA) was passed from the high anterolateral portal and the shaver (Linvatec, Largo, Florida, USA) was inserted through the high anteromedial portal (Fig. 1A). After removing the patellar fat pad and inflamed synovium which blocked the visual field, a large amount of inflamed synovial membrane could be seen (Fig. 1B).
2. Cleaning of synovial membrane in the posterior compartment: arthroscope was passed from the high anterolateral portal, and then advanced posteriorly between the femoral medial condyle and the posterior cruciate ligament. Elevation of the arm, without too much force, could facilitate the advance of the arthroscope along the posterior slope of the tibia into the posterolateral compartment. Then, the arthroscope was withdrawn until the femoral medial condyle falling into the visual field (Fig. 1C). At this position, the arthroscope was turn to point superiorly. Under direct visualization, the high posteromedial portal was established. A needle was inserted anterior to the medial head of the gastrocnemius to avoid neurovascular structures. After proper positioning, a small skin incision was made and a straight clamp was used to separate the soft tissue till reaching the joint capsule. A working approach has been established (Fig. 1D). An exchange rod was inserted through the high posteromedial portal into the posteromedial compartment, and changed to a blunt tip after the arthroscopic sheath was inserted over it. The blunt tip was used to establish a trans-septal portal into the posterior compartment. Finally, the blunt tip was changed to the arthroscopic sheath and the lateral femoral condyle and lateral meniscus could be visualized (Fig. 1E). Posterolateral portal was established and the shaver was inserted similarly under direct visualization of the arthroscopy (Fig. 1F). Finally, the overgrown synovium in the septal, posterior joint capsule, and posteromedial and posterolateral compartments was resected with the arthroscope and shaver through the high posteromedial and posterolateral portals.
3. Cleaning of synovial membrane in the anterior compartment: after completing the posterior synovial membrane, the arthroscope was placed into the high anterolateral portal, with the shaver placed through the high anteromedial portal into the intercondylar fossa (Fig. 2A). The synovial membrane near the cruciate ligament and medial meniscus was resected. In addition, part of the medial meniscus was removed if there was a severe damage to it (Fig. 2B). Then, with legs at the cross position, arthroscope and shaver exchanged their inserting portals and synovial membrane in the anterolateral compartment was removed. Part of the lateral meniscus was removed if there was a severe damage to it (Fig. 2C).
4. Cleaning of synovial membrane in patellar capsule and medial and lateral sulcus: after completing the cleaning in anterior and posterior compartments, arthroscope was placed into the high anterolateral portal. Under the direct visualization of the arthroscope, the shaver was inserted through the superolateral patellar portal after needle positioning (Figs. 2D and 2E). First, inflamed synovium in the patellar capsule was removed. Then, the synovial membrane was cleaned in the medial and lateral sulcus and rest of the patellar capsule with the arthroscope and shaver switching among three different portals (high anterolateral, high anteromedial, and superolateral patellar portals).
5. Postoperative hemostasis and drainage: in patients assigned into the NPD group, vaporized electric knife (Smith & Nephew, London, UK) was used for hemostasis with the tourniquet still in the lower extremity. Then, drainage tube with negative pressure was placed. A compression dressing with gauze and elastic bandage was applied to reduce swelling and provide support for early activities. NPD was commonly used for 1–2 days postoperatively to minimize the intra-articular hematoma, and removed when the drainage was less than 50 ml/day.

Patients in the non-NPD group received the same surgical procedures as the patients in the NPD group, except that, after cleaning of the synovial membrane, vaporized electric knife was used for complete hemostasis in all the compartments after removing the tourniquet (Fig. 2F). There was no drainage tube placed.

Resected synovial tissues were sent for pathology studies. Patients received oral celecoxib and participated into rehabilitation exercise. Meanwhile, instructions were given for limb elevation (higher than the heart) and ankle pump exercise.

### Outcome measurements

Outcomes were health assessment questionnaire (HAQ), disease activity score 28 (DAS 28), and Lysholm knee joint score. HAQ is the gold standard to measure the health status in patients with RA. DAS 28 has been validated in monitoring of RA disease activity in the clinical practice. Lysholm knee joint score has broad applications in evaluating knee joint function and can be completed in a short period of time (Maska, Anderson & Michaud, 2011; Wells et al., 2009; Collins et al., 2011; Wang et al., 2016). These were evaluated before the surgery and at six weeks, three months, and one year after the surgery.

### Statistical analysis

Continuous data were presented as mean ± standard deviation and were compared by student *t*-test. Categorical data presented as percentages and were compared by Chi square analysis. A *P* < 0.05 was considered statistically significantly different. All the statistical analyses were performed with SPSS (version 13.0; IBM, Armonk, New York, USA).

## Results

**Figure 3:**
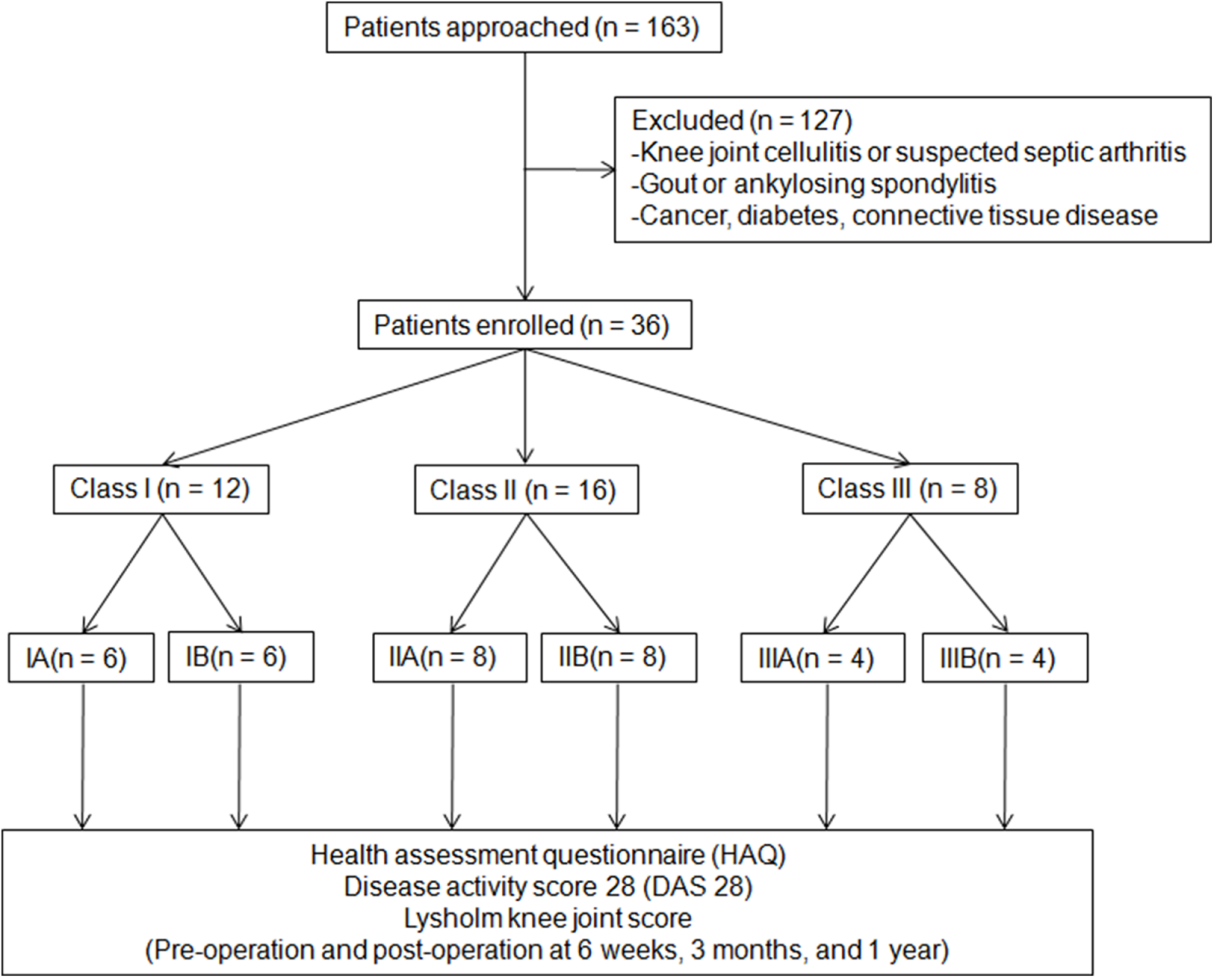
CONSORT flow diagram.

There were 36 patients enrolled into the study, with 63.9% (23) female patients and mean age of 47.2 years old. The number of patients with class II, III, and IV Steinbrocker RAX classification were 12, 16, and eight, respectively. Patient enrollment was shown in Fig. 3. Their baseline characteristics were listed in Table 1. There were no statistically significant differences on these measured baseline characteristics. All study patients successfully completed the arthroscopic synovectomy and were followed up at six weeks, three months, and one year after the operation.

**Table 1:** Baseline characteristics of study participants.

	Steinbrocker RAX classification					
	IIA (*N* = 6)	IIB (*N* = 6)	IIA (*N* = 8)	IIB (*N* = 8)	IIA (*N* = 4)	IIB (*N* = 4)
Age, year, mean ± standard deviation	41.3 ± 7.2	43.2 ± 9.8	45.9 ± 9.9	47.1 ± 8.3	55.8 ± 9.8	56.3 ± 7.7
Gender						
Female, *N* (%)	4 (66.7%)	5 (83.3%)	6 (75.0%)	5 (62.5%)	3 (75.0%)	2 (50.0%)
Affected knee						
Left, *N* (%)	5 (83.3%)	3 (50.0%)	4 (50.0%)	5 (62.5%)	2 (50.0%)	3 (75.0%)

**Notes:**

A, with post-operative negative drainage.

B, without post-operative negative drainage.

**Figure 4:**
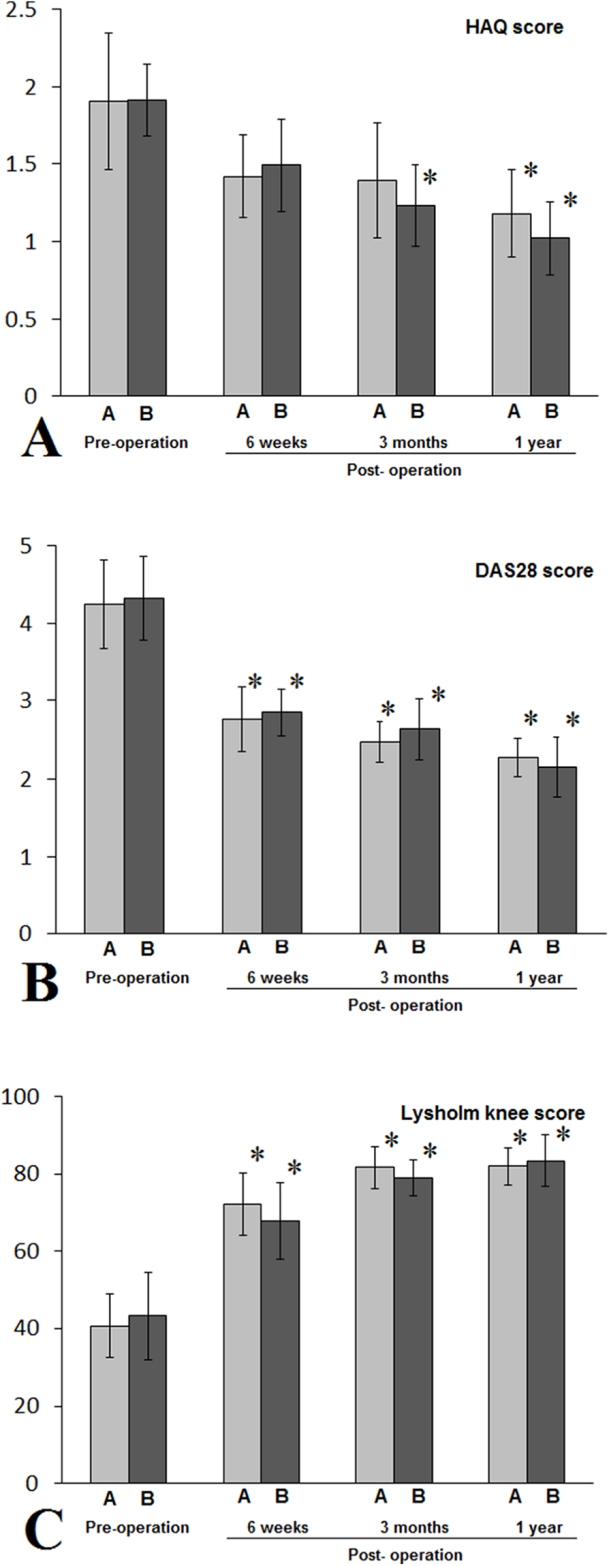
Patients with class I RA in either A (NPD) or B (non-NPD) group were evaluated by HAQ (A), DAS 28 (B), Lysholm knee scores (C). Patients in both A and B groups had statistically significant improvements in all three outcome measurements (*, *P* < 0.05). When compared to pre-operation periods, there were no statistically significant differences between A and B groups during pre-operation period, or six weeks, three months, or one year after the arthroscopic synovectomy treatments. HAQ, health assessment questionnaire; DAS 28, disease activity score 28.

**Figure 5:**
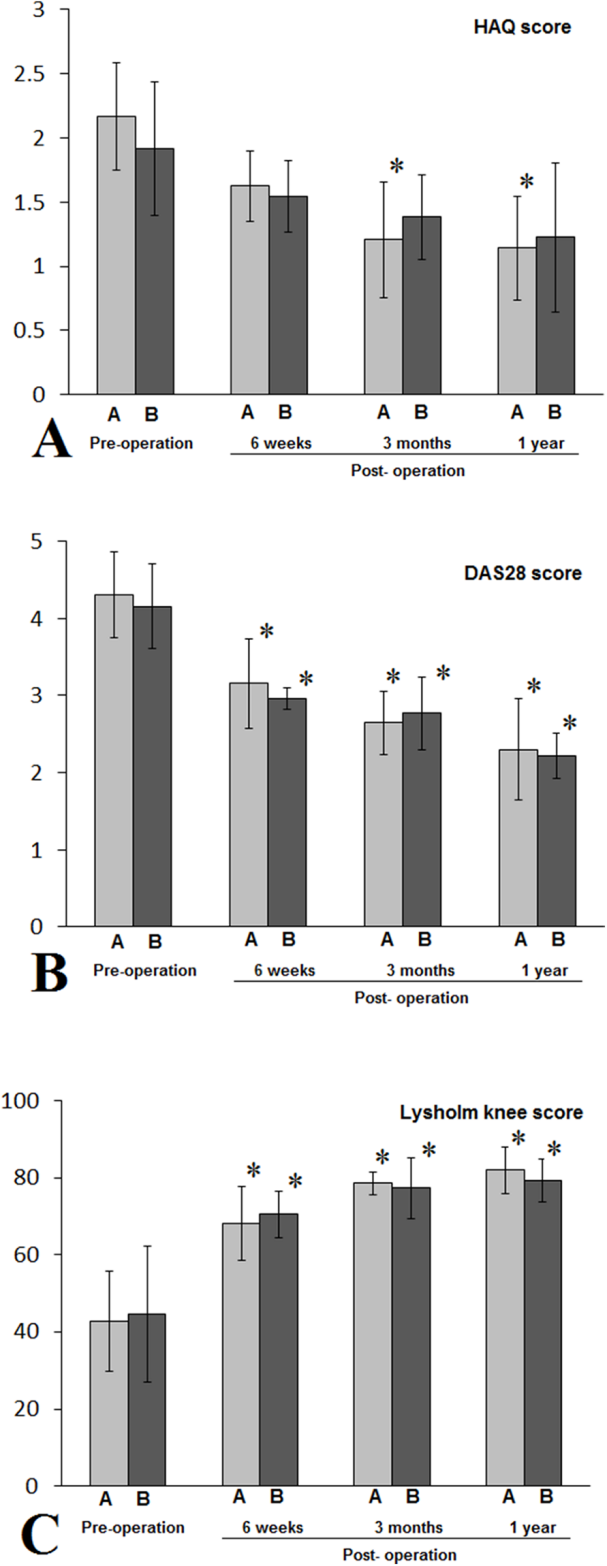
Patients with class II RA in either A (NPD) or B (non-NPD) group were evaluated by HAQ (A), DAS 28 (B), Lysholm knee scores (C). Except patients in IIB and evaluated by HAQ score, patients in both A and B groups had statistically significant improvements in all three outcome measurements (*, *P* < 0.05), when compared to pre-operation periods. There were no statistically significant differences between A and B groups during pre-operation period, or six weeks, three months, or one year after the arthroscopic synovectomy treatments. HAQ, health assessment questionnaire; DAS 28, disease activity score 28.

**Figure 6:**
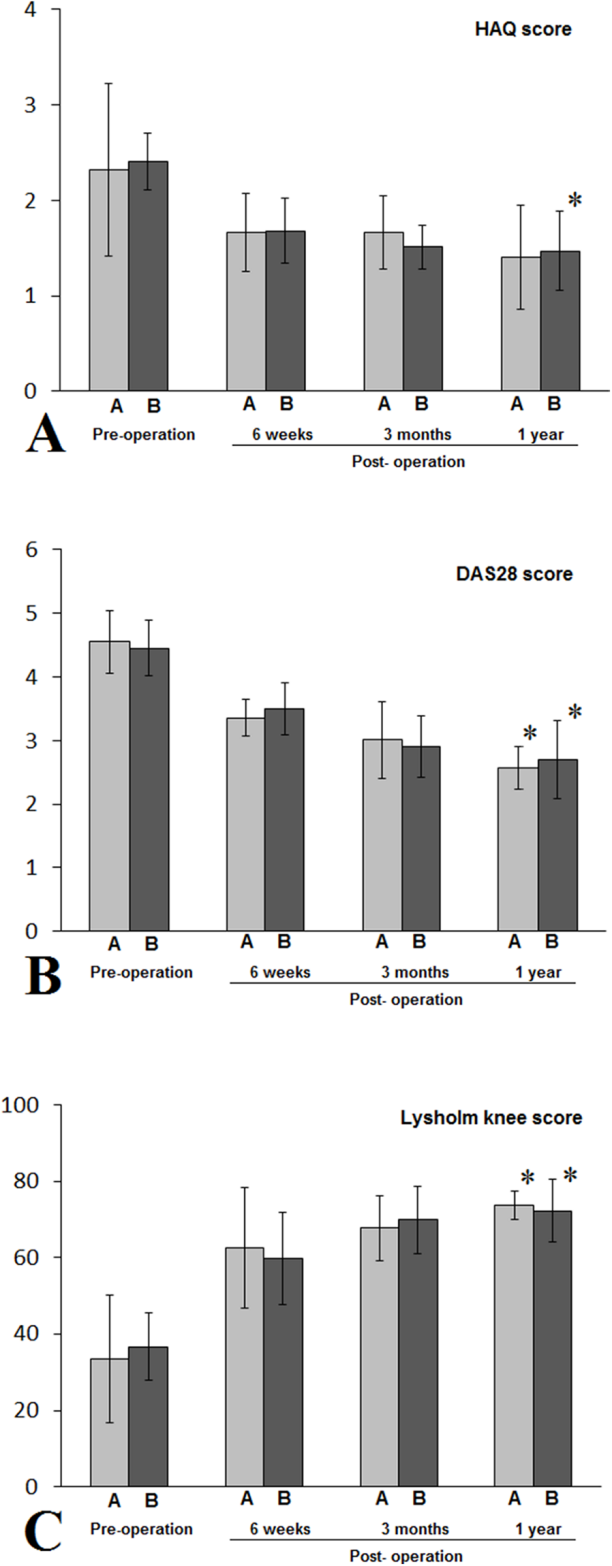
Patients with class III RA in either A (NPD) or B (non-NPD) group were evaluated by HAQ (A), DAS 28 (B), Lysholm knee scores (C). Except patients in IIIA and evaluated by HAQ score, patients in both A and B groups had statistically significant improvements in all three outcome measurements (*, *P* < 0.05), when compared to pre-operation periods. There were no statistically significant differences between A and B groups during pre-operation period, or six weeks, three months, or one year after the arthroscopic synovectomy treatments. HAQ, health assessment questionnaire; DAS 28, disease activity score 28.

All patients successfully completed the arthroscopic synovectomy. In patients with class I RA, all three outcome measurements had statistically significant postoperative changes, with DAS 28 and Lysholm knee score improvements starting to show significant changes at six weeks after the surgery for both groups, and HAQ score improvement started at three months after the surgery for non-NPD group and one year after the surgery for NPD group (Fig. 4). In patients with class II RA, both DAS 28 and Lysholm knee score had statistically significant improvements starting at six weeks after the surgery for both groups, whereas statistically significant improvement in HAQ score was only observed starting from three months after the surgery in NPD group (Fig. 5). In patients with class III RA, statistically significant improvements in DAS 28 and Lysholm knee scores were observed at one year after the surgery for both NPD and non-NPD groups, whereas statistically significant improvement in HAQ score was only seen at year after the surgery for non-NPD group (Fig. 6). There were no statistically significant differences between patients with or without NPD therapy in either class I, II, or III RA in terms of HAQ, DAS 28, and Lysholm knee joint scores.

## Discussion

Rheumatoid arthritis is a common disease. Our current study showed that five portal arthroscopic synovectomy could reduce disease activities, as well as improve knee function and quality of life. Negative pressure drainage had no additional benefits.

Appropriate managements of RA require early recognition and diagnosis. Commonly accepted diagnostic criteria was the 2010 ACR/EULAR classification criteria, which carries high sensitivity and specificity (Raja et al., 2012). Once the diagnosis is established, DMARDs should be prescribed promptly in order to preserve joint integrity and functions. In addition, NSAIDs and corticosteroids are used as the adjunctive therapies to relieve the clinical symptoms. However, a significant number of patients showed poor or no responses to these pharmaceutical treatments. The disease can progress from synovial membrane inflammations, cartilage damages, to bone erosions, and finally, joint destructions (McInnes & Schett, 2011). Affected patients could lose joint functions and have decreased quality of life. Synovectomy was recommended in patients with no responses to the pharmaceutical treatments for six months and not yet meeting indications for joint replacement surgery (Chalmers et al., 2011). It was reported that open synovectomy could provide temporary relief of the joint pain (Paus et al., 1992). With the development of arthroscopic minimally invasive surgery, arthroscopic synovectomy was applied in the treatment for joint involvements in patients with RA. Knee arthroscopy could provide more comprehensive exploration of the knee joint cavity, including the anterior, posterior, and posterolateral compartments of the knee. After identifying the inflamed synovium, arthroscopy could also remove the synovial lesions and control the further development of synovitis. Studies have shown that arthroscopic synovectomy could result in prolonged symptom relief and low recurrence rate with less surgical damage and decreased cost (Kanbe et al., 2015; Ogilvie-Harris & Weisleder, 1995; Gibbons, Gosal & Bartlett, 2002; Roch-Bras et al., 2002). It could also avoid the long-term complications from pharmaceutical therapy, such as gastrointestinal reactions and liver toxicity.

Traditionally, arthroscopic synovectomy could be performed with either five or six portal approach. Five portal approach only requires to make five small incisions around the knee joint and causes less injury than the six portal approach. In the current study, we have demonstrated that five portal arthroscopy could provide adequate visualization and exploration of anterior, posterior, and posterolateral compartments of the knee joint. All the study patients have successfully completed the surgical operation and their inflamed synovial lesions were removed. All the study patients were also followed up at six weeks, three months, and one year after the surgery. Patients had statistically significantly better qualities of life, reduced disease activities, and improved knee joint functions, suggesting that, in patients with rheumatoid knee arthritis not seriously enough for knee replacement surgery, five portal arthroscopic synovectomy could improve knee joint activity and functions. Benefits on disease activity reduction and knee joint function were observed at six weeks postoperatively, and improvement of quality of life happened at one year after the surgery. These benefits were observed more in patients with class I or II RA but not class III RA, which might suggest that arthroscopic synovectomy should be performed earlier in the disease development in order to save the joint and improve the quality of life.

There were controversies on whether post-operation NPD was necessary after joint surgery (Siqueira et al., 2016; Karlakki et al., 2013; Clifton et al., 2007). Our current study showed no differences in terms of quality of life, disease activities, and knee joint functions between patients with or without NPD after the arthroscopic synovectomy. This was consistent with the previous report by Clifton, who performed a meta-analysis showing no benefits for NPD after knee surgery (Clifton et al., 2007). The proposed underlying therapeutic effect of NPD was to remove joint blood collection, which was believed to stimulate synovium and increase inflammation after the surgery (Johnson, 1991). As long as the post-operation bleeding is controlled, NPD may not be necessary.

Limitations of the current study included small sample size and single-center study with short-term follow-up time. We also did not include a controlled group, although all the enrolled patients had poor responses to the traditional pharmaceutical therapy for the previous six months. According to a previous publication, small number of patients could experience adverse events after arthroscopic synovectomy. In the current clinical trial with a small number of patients, we did not investigate the adverse events after the surgical procedure. Further randomized control clinical trials with a large sample size and long follow-up time are required to confirm the efficacy and investigate the adverse events with this surgical approach.

In conclusion, five portal arthroscopic synovectomy could effectively increase the quality of life, decrease disease activities, and improve joint functions in patients with rheumatoid arthritis of the knee. Post-operation NPD did not show additional benefits.

## Supplemental Information

10.7717/peerj.4727/supp-1 Supplemental Information 1 Raw data.

10.7717/peerj.4727/supp-2 Supplemental Information 2 Study protocol.

10.7717/peerj.4727/supp-3 Supplemental Information 3 CONSORT checklist.

## Additional Information and Declarations

### Data Availability

The following information was supplied regarding data availability: The raw data has been supplied as a Supplemental File.

### Clinical Trial Registration

The following information was supplied regarding Clinical Trial registration: Chinese Ethics Committee of Registering Clinical Trials: chiCTR-IPQ-17012440.
