# Supplementary material for: Improved quality of life and joint functions in patients with knee rheumatoid arthritis who underwent five portal arthroscopic synovectomy
Source: PeerJ. 2018 Apr 30;6:e4727. doi: 10.7717/peerj.4727 (PMC5933344; doi:10.7717/peerj.4727)
Supplement: Supplemental Information 2 [file peerj-06-4727-s002.docx]

**Study protocol**

After informed consent process, patients were classified into class I, II, and III based on the RAX Steinbrocker classification system. Then, patients were further assigned into either A (NPD) or B (non-NPD) group according to the even or odd number of their ordered admission numbers.

All the patients received the surgical operations under the laryngeal mask general anesthesia with regional nerve (femoral nerve and sciatic nerve) blocks by one orthopedic physician. With patients in the supine position and affected leg close to the edge of the bed, cotton-lined tourniquet was placed to the thigh proximal to the affected knee. Two arthroscopic pads were placed at the lateral side of the distal thigh (close to the lateral side of joint line) and middle thigh, in order to ensure that the leg could be easily placed with a slight valgus overhanging position and knee could be placed at 90 degree flexion position. After standard sterilization and applying dressings to the lifted leg, the tourniquet was inflated to a pressure of 400 – 600 mmHg. A complete synovial resection usually required at least five portal approaches. Thus, in the current study, we used five portal approaches (high anterolateral approach, high anteromedial approach, high posterior medial approach, posterior lateral approach, and superolateral patellar approach) in order to completely resect the knee synovium.

The surgical procedures were:

1. Initial exploration of knee joint: arthroscope (Linvatec, United States) was passed from the high anterolateral portal and the shaver (Linvatec, United States) was inserted through the high anteromedial portal (Figure 1A). After removing the patellar fat pad and inflamed synovium which blocked the visual field, a large amount of inflamed synovial membrane could be seen (Figure 1B).
2. Cleaning of synovial membrane in the posterior compartment: arthroscope was passed from the high anterolateral portal, and then advanced posteriorly between the femoral medial condyle and the posterior cruciate ligament. Elevation of the arm, without too much force, could facilitate the advance of the arthroscope along the posterior slope of the tibia into the posterolateral compartment. Then, the arthroscope was withdrawn until the femoral medial condyle falling into the visual field (Figure 1C). At this position, the arthroscope was turn to point superiorly. Under direct visualization, the high posteromedial portal was established. A needle was inserted anterior to the medial head of the gastrocnemius to avoid neurovascular structures. After proper positioning, a small skin incision was made and a straight clamp was used to separate the soft tissue till reaching the joint capsule. A working approach has been established (Figure 1D). An exchange rod was inserted through the high posteromedial portal into the posteromedial compartment, and changed to a blunt tip after the arthroscopic sheath was inserted over it. The blunt tip was used to establish a trans-septal portal into the posterior compartment. Finally, the blunt tip was changed to the arthroscopic sheath and the lateral femoral condyle and lateral meniscus could be visualized (Figure 1E). Posterolateral portal was established and the shaver was inserted similarly under direct visualization of the arthroscopy (Figure 1F). Finally, the overgrown synovium in the septal, posterior joint capsule, and posteromedial and posterolateral compartments was resected with the arthroscope and shaver through the high posteromedial and posterolateral portals.
3. Cleaning of synovial membrane in the anterior compartment: after completing the posterior synovial membrane, the arthroscope was placed into the high anterolateral portal, with the shaver placed through the high anteromedial portal into the intercondylar fossa (Figure 2A). The synovial membrane near the cruciate ligament and medial meniscus was resected. In addition, part of the medial meniscus was removed if there was a severe damage to it (Figure 2B). Then, with legs at the cross position, arthroscope and shaver exchanged their inserting portals and synovial membrane in the anterolateral compartment was removed. Part of the lateral meniscus was removed if there was a severe damage to it (Figure 2C).
4. Cleaning of synovial membrane in patellar capsule and medial and lateral sulcus: after completing the cleaning in anterior and posterior compartments, arthroscope was placed into the high anterolateral portal. Under the direct visualization of the arthroscope, the shaver was inserted through the superolateral patellar portal after needle positioning (Figure 2D, 2E). First, inflamed synovium in the patellar capsule was removed. Then, the synovial membrane was cleaned in the medial and lateral sulcus and rest of the patellar capsule with the arthroscope and shaver switching among three different portals (high anterolateral, high anteromedial, and superolateral patellar portals).
5. Postoperative hemostasis and drainage: in patients assigned into the NPD group, vaporized electric knife (Smith & Nephew, United Kingdom) was used for hemostasis with the tourniquet still in the lower extremity. Then, drainage tube with negative pressure was placed. A compression dressing with gauze and elastic bandage was applied to reduce swelling and provide support for early activities. NPD was commonly used for 1 – 2 days postoperatively to minimize the intraarticular hematoma, and removed when the drainage was less than 50 ml/day.

Patients in the non-NPD group received the same surgical procedures as the patients in the NPD group, except that, after cleaning of the synovial membrane, vaporized electric knife was used for complete hemostasis in all the compartments after removing the tourniquet (Figure 2F). There was no drainage tube placed.

Resected synovial tissues were sent for pathology studies. Patients received oral celecoxib and participated into rehabilitation exercise. Meanwhile, instructions were given for limb elevation (higher than the heart) and ankle pump exercise.

Outcome measurements

Outcomes were health assessment questionnaire (HAQ), disease activity score 28 (DAS 28), and Lysholm knee joint score. These were evaluated before the surgery and at 6 weeks, 3 months, and 1 year after the surgery.

Statistical analysis

Continuous data were presented as mean ± standard deviation and were compared by student t-test. Categorical data presented as percentages and were compared by Chi square analysis. A *P* < 0.05 was considered statistically significantly different. All the statistical analyses were performed with SPSS (version 13.0, IBM, United States).
